# Immune4Plasticity: do non-pharmacological interventions modulate the inflammatory pattern of major depressive disorder? A study protocol

**DOI:** 10.1192/j.eurpsy.2025.757

**Published:** 2025-08-26

**Authors:** M. Di Vincenzo, B. Della Rocca, C. Toni, I. Branchi, F. Cirulli, S. Poletti, S. Poggini, A. Viglione, C. Delli Colli, C. Musillo, M. Luciano, G. Sampogna, A. Fiorillo

**Affiliations:** 1 Department of Psychiatry, University of Campania “Luigi Vanvitelli”, Naples; 2 Center for Behavioral Sciences and Mental Health; 3 Section of Behavioral Neuroscience, Department of Cell Biology and Neurosciences, Istituto Superiore di Sanità, Rome; 4 Psychiatry and Clinical Psychobiology, Division of Neuroscience, Vita-Salute San Raffaele University, Milan, Italy

## Abstract

**Introduction:**

Major depressive disorder (MDD) is a severe mental disorder with a prevalence rate of 10%. Approximately 30-40% of patients suffering from MDD show higher levels of proinflammatory cytokines, associated to low response to pharmacotherapy. Thus, modulation of immune system might have a key role in the management of MDD.

**Objectives:**

This study is aimed to: 1) assess the interrelation between immune hyperactivation and neuronal plasticity; 2) assess how non-pharmacological treatments impact on the immune hyperactivity in patients suffering from MDD; 3) identify biological makers able to predict the course of MDD and the effectiveness of treatments.

**Methods:**

Immune4Plasticity is a longitudinal, multisite trial funded by Italian Ministry of Health. Preclinical analyses aimed at investigating the interrelation between immune hyperactivation and neuroplasticity as well as the identification of biological markers of MDD will be carried out at National Institute of Health in Rome. Clinical part of the study will be performed at the Department of Psychiatry of University of Campania, Naples, and at the Vita-Salute San Raffaele University, Milan. Seventy patients aging 18-65, with a diagnosis of MDD according to the DMS-5 criteria without psychotic symptoms, scoring more than 14 at the 17-item Hamilton Depression Rating Scale and able to release informed consent will be included. Thirty-five participants will attend a lifestyle psychosocial intervention in Naples; thirty-five will undergo light-therapy sessions in Milan. Assessments of both groups will be performed at recruitment (T0), after 3 months (T1) and after 6 months (T2), by using standardized psychometric tools and blood samples. The project will be carried out for 24 months.

**Results:**

This multidisciplinary, translational study will shed more light on the complex interrelationship between MDD, immune system and neuroplasticity by investigating the role of psychosocial intervention and light therapy as ‘modulators’. This will make it possible to develop innovative therapeutic strategies by integrating non-pharmacological approaches with anti-inflammatory drugs and to identify new peripheral markers to assess the response to treatment of patients with MDD.

**Conclusions:**

MDD is a complex mental disorder associated with higher expression of inflammation. Sometimes, it is not adequately responsive to pharmacotherapy. Understanding the effect of non-pharmacological treatments as “modulators” of the inflammatory pattern of MDD may be an important strategy to optimize clinical management of this disorder.

**Disclosure of Interest:**

None Declared

